# Frontal–striatal–thalamic circuit dysfunction and inferential processing between internal representations and external events across the psychosis spectrum

**DOI:** 10.3389/fpsyt.2026.1930683

**Published:** 2026-09-29

**Authors:** Naoyuki Katagiri, Hiromi Tagata, Takashi Uchino, Yu Arai, Karin Hayashi, Junichi Saito, Kouhei Kamiya, Masaaki Hori, Masafumi Mizuno, Takahiro Nemoto

**Affiliations:** 1 Department of Neuropsychiatry, Sakura Medical Center, Faculty of Medicine, Toho University, Sakura, Chiba, Japan; 2 Department of Neuropsychiatry, Toho University School of Medicine, Ota-ku, Tokyo, Japan; 3 Department of Psychiatry and Implementation Science, Toho University School of Medicine, Ota-ku, Tokyo, Japan; 4 Department of Radiology, Toho University School of Medicine, Ota-ku, Tokyo, Japan; 5 Asaka Hospital, Koriyama, Fukushima, Japan

**Keywords:** at-risk mental states, attenuated psychosis syndrome, frontal–striatal–thalamic circuit, inferential processing, phenomenology, subjective consciousness

## Abstract

**Background:**

In psychopathology, schizophrenia has been understood as a disorder characterized by disturbances in subjective consciousness. Subjective consciousness and its relationship to interactions between internal representations and external events have been discussed in phenomenology. Recent computational neuroscience conceptualizes the brain as an inferential system that minimizes discrepancies between internal models and latent states inferred from environmental information. From a neurobiological perspective, the frontal–striatal–thalamic circuit may underlie the inferential processing involved in updating internal models based on sensory information derived from the environment.

**Aim:**

In this review, we aimed to examine the theoretical point of contact between phenomenology and computational neuroscience from the perspective of inferential processing within the frontal–striatal–thalamic circuit. An imbalance within this circuit may disrupt inferential processes that minimize discrepancies between internal models and latent states. Such maladaptive inferential processes may distort subjective experiences, potentially contributing to the symptoms observed in At-Risk Mental States (ARMS) and Attenuated Psychosis Syndrome (APS), which are considered clinically high-risk states for psychosis. Based on these considerations, we propose a model linking psychopathological phenomena and neurobiological alterations through the dysfunction of the frontal–striatal–thalamic circuit and associated disturbances in inferential processing. We assessed whether this model was theoretically consistent with previous findings and our own longitudinal studies on ARMS/APS.

**Conclusions:**

This conceptual model may provide a novel perspective on the information-processing mechanisms, particularly inferential processing, and their alterations across high-risk clinical states for psychosis, including ARMS/APS and broader Clinical High At Risk Mental States (CHARMS) concept.

## 1 Introduction

Jaspers (1) conceptualized schizophrenia as a pathological condition based on disturbances in self-consciousness (German: *Ichbewusstsein*). Disturbances in self-consciousness destabilize object consciousness, which exists in a complementary relationship, thereby disrupting the overall equilibrium of subjective consciousness. Therefore, elucidating the processes underlying the emergence of subjective consciousness is indispensable for understanding the pathogenesis of schizophrenia. However, because subjective and objective phenomena exist in different dimensions, there are inherent theoretical limitations in explaining subjective consciousness solely through objective indicators. This remains a fundamental dilemma between clinical psychiatry and neuroscience.

Nevertheless, attempts have been made to explore the theoretical point of contact between subjective consciousness and neuroscience. Subjective consciousness and its relationship to interactions between internal representations and external events have been discussed in phenomenology, while computational neuroscience conceptualizes the brain as an inferential system that minimizes discrepancies between internal models and latent states (2). These two perspectives may converge when viewing reciprocal interactions between internal representations and environment-derived information as a common theoretical basis. From a neurobiological perspective, the frontal–striatal–thalamic circuit may underlie the inferential processing involved in updating internal models based on sensory information derived from the environment. Alterations in the equilibrium within this circuit are hypothesized to disturb the inferential processes involved in resolving discrepancies between internal representations and external events, thereby contributing to changes in subjective experience (**Figure 1**).

**Figure 1:**
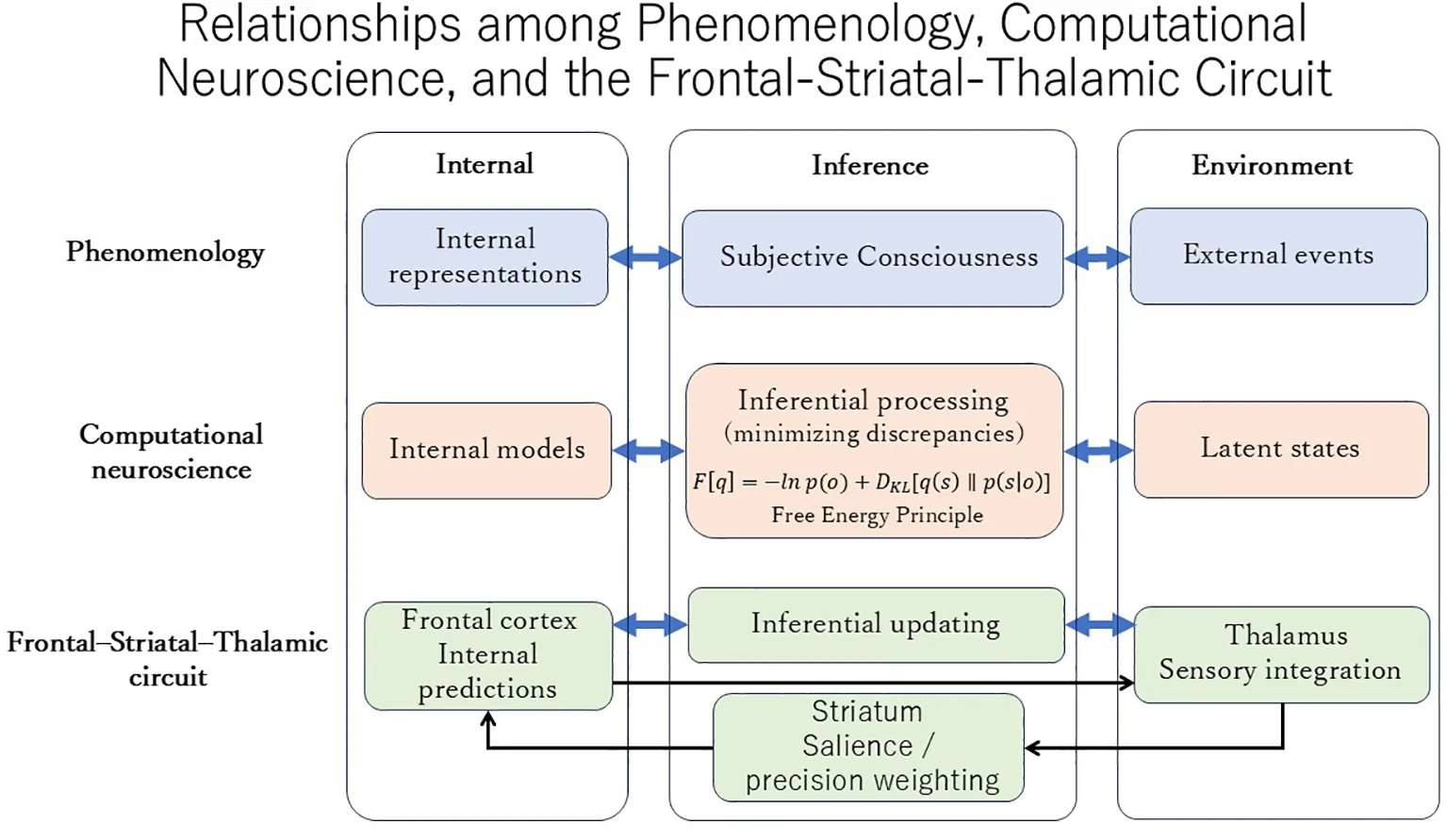
Relationships among phenomenology, computational neuroscience, and the frontal–striatal–thalamic circuit. This conceptual diagram illustrates the theoretical relationships among phenomenology, computational neuroscience, and the frontal–striatal–thalamic circuit. In the working definition adopted in the present review, subjective consciousness is considered a phenomenon involving interactions between internal representations and external events. Computational neuroscience conceptualizes the brain as an inferential system that minimizes discrepancies between internal models and latent states based on the free-energy principle. The frontal–striatal–thalamic circuit is proposed as one potential neural substrate underlying inferential updating between internal predictions and environment-derived sensory information integrated by the thalamus.

From a psychopathological perspective, subtle disturbances in mental functioning including subjective experience precede the onset of overt psychosis. Accordingly, At-Risk Mental States (ARMS) and Attenuated Psychosis Syndrome (APS) may represent clinically observable stages in which subtle alterations in inferential processing and subjective experience begin to emerge before the development of schizophrenia (3). Therefore, these states provide a unique opportunity to investigate how biological alterations relate to the emergence of psychopathological disturbances. In this review, we focus on ARMS/APS to examine whether biological alterations within the frontal–striatal–thalamic circuit are theoretically consistent with disturbances in inferential processing that may underlie emerging changes in subjective experience.

## 2 Methods

This narrative review focuses on the reciprocal interactions between internal representations and the external environment and examines their theoretical relationships to phenomenology and computational neuroscience. Particular attention was given to the frontal–striatal–thalamic circuit as a potential neural substrate underlying inferential processing. A schematic overview of these theoretical relationships is presented in **Figure 1**. To examine this conceptual model, the review aimed to identify theoretical relationships and potential points of contact across different academic fields, including phenomenology, psychopathology, neuroscience, and computational neuroscience, regarding the mechanisms underlying the emergence of subjective consciousness and its disturbances.

These fields differ substantially in their methodological foundations, ranging from deductive and conceptual approaches in phenomenology and psychopathology to predominantly empirical and inductive approaches in neuroimaging and clinical research. Therefore, applying a single search strategy and uniform criteria for literature selection across all of these fields has inherent limitations. Accordingly, literature was selected using approaches appropriate to the methodological characteristics of each field. No specific publication-date restriction was applied, as the interdisciplinary scope of this review involved tracing theoretical concepts to their historical origins while also incorporating recent empirical evidence.

For phenomenology and psychopathology, major original works and representative literature providing the theoretical foundations for the concepts discussed in this review were selected. To examine empirical support for these theoretical relationships, literature in neuroscience, computational neuroscience, and clinical research was searched primarily through PubMed, with additional relevant studies identified from the reference lists of key publications. In these fields, representative reviews, large-scale meta-analyses, and multicenter studies were prioritized.

Findings from our previous longitudinal neurobiological studies investigating the frontal–striatal–thalamic circuit in ARMS/APS were also incorporated as individual studies relevant to the proposed conceptual model. Because these studies were conducted at a single center and generally involved modest sample sizes, their consistency with the broader literature, including independent studies published before and after our reports, was examined to assess the extent to which they were supported by convergent evidence and to identify their limitations. A summary of these theoretical relationships and representative evidence is presented in **Table 1**.

**Table 1:** Points of contact among major theoretical concepts and representative evidence linking phenomenology, inferential processing, and the frontal–striatal–thalamic circuit.

Major concept or evidence	Core proposition	Proposed relevance to the frontal–striatal–thalamic model	Evidence type	Key representative literature
Phenomenology / subjective consciousness	Subjective consciousness can be understood as experience emerging through the relationship between internal representations and external events. Disturbances of self-consciousness/minimal self are features of schizophrenia psychopathology.	Provides a phenomenological foundation for the model by linking subjective experience to interactions between internal representations and external events (**Figure 1**).	Phenomenology / psychopathology	Husserl (1913) (6); Jaspers (1913) (1); Sass & Parnas (2003) (5); Nelson et al. (2014) (59, 60)
Bayesian inference / predictive processing / free-energy principle	The brain integrates prior predictions derived from internal models with sensory information to infer latent states of the environment. Prediction-error-dependent updating continuously shapes perception and action.	Provides a mathematical and computational formulation of inferential processing between internal models and latent states (**Figure 1**).	Computational / theoretical	Helmholtz (1867) (7); Friston (2010) (2); Knill & Pouget (2004) (8); Friston et al. (2017) (12); Fletcher & Frith (2009) (20)
Frontal–striatal–thalamic circuit / active inference	Cortico–striato–thalamo–cortical systems comprise multiple interacting parallel subcircuits and contribute to inferential updating through perception and action.	Positions the frontal–striatal–thalamic circuit as one potential anatomical substrate supporting reciprocal interactions between internal predictions and integrated sensory information during inferential processing (**Figure 1**).	Systems neuroscience / theoretical	Friston et al. (2017) (12); Alexander et al. (1986) (13); Haber (2016) (15)
Aberrant salience / striatal dopamine	Striatal dopamine contributes to salience attribution, and psychosis-related dopaminergic abnormalities are particularly prominent in the associative/dorsal striatum. Aberrant dopaminergic signaling may alter salience/precision weighting.	Provides a biological mechanism linking dopaminergic salience in the striatum to abnormal weighting of information within frontal–striatal–thalamic processing.	Dopamine PET / theoretical	Kapur (2003) (18); Howes et al. (2009) (44); Egerton et al. (2013) (45); McCutcheon et al. (2019) (46)
Strong- and weak-prior hypotheses / hierarchical inference	Psychosis has been associated with both excessive weighting of prior predictions (strong priors) and reduced precision of priors (weak priors). These abnormalities may occur at different levels of the inferential hierarchy.	Integrates apparently opposing strong- and weak-prior findings as abnormalities of precision weighting at different hierarchical levels and provides testable computational hypotheses for the frontal–striatal–thalamic model.	Computational + behavioral	Powers et al. (2017) (22); Nassar et al. (2021) (23); Takei et al. (2026) (24); Sterzer et al. (2018) (25); Adams et al. (2013) (26)
Large-scale neuroimaging evidence relevant to frontal, striatal, and thalamic systems	Large-scale studies and meta-analyses in schizophrenia/CHR have reported structural abnormalities involving frontal regions and white-matter pathways, striatal/subcortical structures, and thalamic regions relevant to the systems considered in this review.	Places the authors’ ARMS/APS findings within independent large-scale and meta-analytic evidence relevant to frontal, striatal, thalamic, and connecting white-matter systems, without implying that these studies are specific to the frontal–striatal–thalamic model.	Meta-analysis / multicenter neuroimaging	Fusar-Poli et al. (2011) (27); Kelly et al. (2018) (28); Merritt et al. (2021) (29); van Erp et al. (2016) (36)
Prediction-error / active-sampling biomarkers	MMN provides an observable index of prediction-error processing, whereas saccades can reflect active sampling that tests perceptual hypotheses.	Links inferential processing, which cannot be directly measured by structural imaging alone, to testable behavioral and neurophysiological measures.	EEG / behavioral / neuroimaging	Bodatsch et al. (2011) (51); Erickson et al. (2016) (52); Friston et al. (2012) (56)

This table summarizes the major theoretical points of contact and representative evidence considered in this review across phenomenological accounts of subjective experience, computational models of inferential processing, and the frontal–striatal–thalamic circuit. The cited studies are representative rather than exhaustive. Large-scale neuroimaging studies are restricted here to findings relevant to frontal, striatal, thalamic, and connecting white-matter systems discussed in the present review. CHR, clinical high risk; ARMS, at-risk mental state; APS, attenuated psychosis syndrome; MMN, mismatch negativity.

## 3 Bridging inductive and deductive approaches

Schizophrenia is considered to develop through long-term interactions among biological vulnerabilities originating in the fetal period, psychological factors, and social factors, which become superimposed and mutually intertwined over time (4). Among these three factors, biological and social factors can be more readily reduced to objective indicators, and substantial evidence has accumulated through inductive approaches based on empirical science. In contrast, psychological factors belong to the dimension of subjective phenomena. Therefore, there are inherent difficulties in reducing psychological factors to the same objective dimension as biological and social factors, and in directly analyzing them through inductive methods. Owing to these limitations, many explanations of the relationship between subjective consciousness, which constitutes the core of psychological factors, and biological or social factors remain at a hypothetical level (5).

Husserl (6) discussed the theoretical limitations of explaining subjective experiences solely through inductive approaches based on objective indicators. Drawing on mathematics and logic, he examined subjective consciousness by focusing on the intentional relationship between consciousness and the objects of experience.

The relationship between subjective consciousness and the experienced world has remained a fundamental concern in phenomenology, providing a theoretical foundation for considering how interactions between internal representations and external events contribute to subjective consciousness. Through the work of Jaspers, phenomenology profoundly influenced the development of modern psychopathology. For the purposes of the present review, we therefore adopt a working definition of subjective consciousness as a phenomenon arising through interactions between internal representations and external events.

From a biological perspective, Helmholtz (7) argued that among the vast amounts of sensory stimuli received from the environment through sensory organs, only limited fragmentary information ascends to the brain, where it is reconstructed through unconscious inference and appears within subjective consciousness as perception. This view conceptualizes perception, which constitutes part of subjective consciousness, as a phenomenon arising through inferential reconstruction of fragmentary information derived from the environment. Accordingly, subjective consciousness can be understood as involving inferential processing. From this perspective, inferential processing provides a theoretical point of contact between phenomenological accounts and mathematical models of inference. This view is conceptually compatible with contemporary computational theories of perception, which describe perception as the outcome of inferential processes that integrate prior expectations with sensory information.

## 4 Bayesian inference and the emergence of computational neuroscience

A theoretical point of contact can be identified between phenomenological accounts of reciprocal interactions between internal representations and external events and Helmholtz’s concept of unconscious inference. Both perspectives converge on the notion of inferential processes. A representative mathematical formulation of such inferential processes is Bayes’ theorem. Bayes’ theorem describes how the probability of a hidden or latent state is updated based on an observation and is expressed as follows:


$$p(s|o)=\frac{p(o|s)p(s)}{p(o)}$$

Knill and Pouget (8) applied Bayesian inference to models of neural information processing by defining latent states *s* as hidden states of the external environment and observations *o* as sensory inputs corresponding to those states. They proposed the Bayesian brain hypothesis, in which the brain’s internal model is conceptualized as a prior distribution updated toward a more plausible posterior distribution through the integration of incoming sensory inputs. This Bayesian inferential perspective subsequently evolved into more comprehensive theories and was further systematized through the free-energy principle (**Figure 1**).

## 5 Inferential processing in the free-energy principle

Building on Bayesian models of inference, Friston (2) conceptualized living organisms as self-organizing systems that maintain homeostasis under environmental uncertainty through inferential processes. This framework was further formalized through the free-energy principle, which was derived from the thermodynamic concept of free energy and is expressed by the following equation:


F=U−TS

(*F*: free energy, *U*: internal energy, *T*: thermodynamic temperature, *S*: entropy).

Subsequently, Friston proposed variational free energy, a concept that integrates thermodynamic free energy with the Bayesian brain hypothesis. The variational free energy is expressed as follows (9):


$$F[q]=-ln\;p(o)+D_{KL}[q(s)∥p(s|o)]$$

(*o*: observation, *s*: latent state of the external environment, *q*(s): recognition density, D*_KL_*: Kullback–Leibler divergence).

The first term, −lnp(o), corresponds to surprisal, defined as the negative logarithm of the probability of an observation, whereas the second term, $D_{KL}[q(s)∥p(s|o)]$ represents the divergence between the recognition density q(s) and the true posterior distribution p(s|o). Therefore, variational free energy F[q] can be interpreted as an index reflecting the degree of mismatch between internal models and latent states inferred from sensory inputs derived from the external environment.

This equation formalizes the process by which living organisms update their recognition density q(s) in a direction that minimizes variational free energy, thereby enabling the internal model to progressively approximate the structure of the external environment. In this formulation, adaptive interactions with the external environment are maintained by minimizing the mismatches between internal predictions and sensory inputs. The resulting inferential process is theoretically consistent with the phenomenological view that subjective consciousness emerges through reciprocal interactions between internal representations and external events (**Figures 1**, **2**).

**Figure 2:**
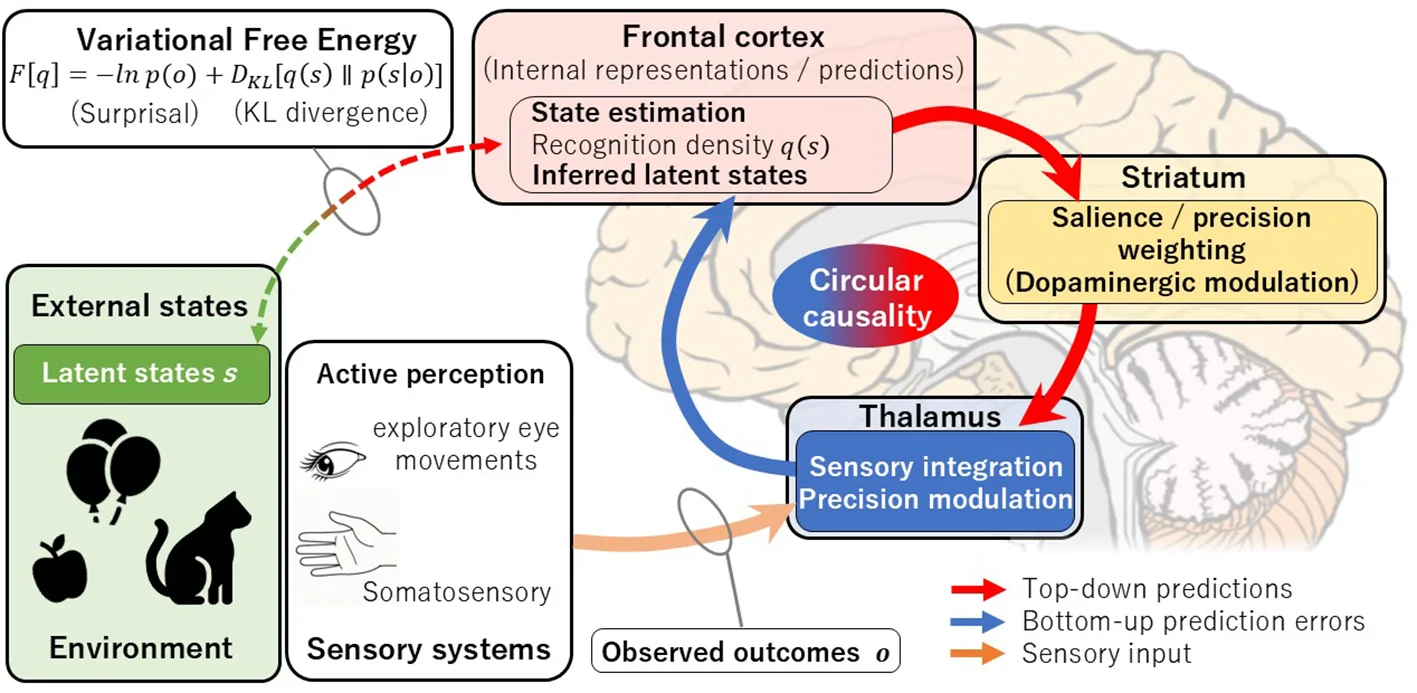
Schematic representation of inferential processing between internal representations and latent states in the frontal–striatal–thalamic circuit. In this conceptual model, the latent states of the external environment are sampled as observed outcomes through sensory systems. The frontal cortex encodes the inferences about latent states through the recognition density *q*(s). Within the frontal–striatal–thalamic circuit, top-down predictions and bottom-up prediction errors interact to support inferential updating. Reciprocal interactions between top-down predictions and bottom-up prediction errors are conceptualized as circular causality. Within this circuit, the striatum modulates salience attribution and precision weighting via dopaminergic signaling. Active perceptual sampling, including exploratory eye movements and perception–action coupling, is illustrated as one form of inferential processing. Variational free energy is schematically represented as the sum of surprisal and the KL divergence between the recognition density *q*(s) and the posterior distribution over latent states given observed outcomes.

## 6 Inferential processing in the frontal–striatal–thalamic circuit

Building on the free-energy principle, inferential processing may be understood as a self-organizing process through which living systems maintain adaptive low-entropy states under environmental uncertainty. Repeated updating of the recognition density q(s) refines inferential processing and enables the internal model to progressively approximate the latent states of the environment. At the perceptual level, this inferential updating can be understood as a process that minimizes discrepancies between predictions and sensory inputs (10).

In predictive processing models, top-down predictive signals generated at higher hierarchical levels descend to lower levels where prediction errors emerge from discrepancies between predictions and actual sensory inputs. These prediction errors are propagated bottom-up and drive the updating of the internal model. Reciprocal interactions between top-down predictions and bottom-up prediction errors are conceptualized as circular causality (11).

Friston et al. (12) further proposed that this updating process is not limited to passive sensory processing, but also incorporates sensory information acquired through active movements of the organism. In other words, perception and action are integrated as inseparable processes that continuously update the internal model through reciprocal interactions between the top-down predictions and bottom-up prediction errors. The integrated process of perception and action is referred to as active inference. Within active inference, inferential processing encompasses state estimation, action selection, and precision estimation. These processes are conceptually separable but interact with one another.

At the neural systems level, various large-scale neural circuits contribute to the integration and processing of information underlying cognition and behavior, including parallel cortico–striato–thalamo–cortical loops and cortico–cerebellar–thalamo–cortical circuits (13–15). Among many regions, cortical regions play a major role in generating top-down predictive signals underlying inferential processing. A substantial amount of sensory information derived from the external environment also converges on the thalamus before being distributed to the cerebral cortex. Recent evidence indicates that higher-order thalamic nuclei, particularly the mediodorsal nucleus, contribute to integrating top-down predictions with bottom-up sensory information and to precision weighting. Structural and functional abnormalities of the mediodorsal thalamus have also been reported in schizophrenia (16, 17), supporting an active role of the thalamus in inferential processing.

Given the respective roles of the frontal cortex in generating top-down predictions and the thalamus in integrating sensory information, we focused on neural circuits linking these two regions. One of the major circuits linking these regions is the frontal–striatal–thalamic circuit. We therefore consider this circuit one potential neurobiological substrate of inferential processes that minimize variational free energy, enabling internal models to progressively approximate the structure of the external environment.

The striatum functions as a hub that regulates and processes information circulating between the frontal lobe and the thalamus. Kapur (18) argued that salience is assigned to information through dopaminergic signaling within the striatum, thereby regulating the priority of information processing circulating between the frontal lobe and the thalamus. On this basis, he proposed the aberrant salience hypothesis, which conceptualizes schizophrenia as a pathological condition arising from abnormalities in salience attribution. Furthermore, Peters et al. (19) suggested that dysfunction in salience processing may be observed not only in schizophrenia but also across a wide range of psychiatric disorders. Accordingly, we examine dysfunction within the frontal–striatal–thalamic circuit as a potential neurobiological substrate of inferential processing between internal representations and external events across the psychosis spectrum (**Figure 2**).

## 7 Internal representations and external events in relation to strong- and weak-prior hypotheses

The frontal cortex is responsible for generating higher-order internal predictions, while the thalamus receives and integrates sensory information derived from the external environment. The striatum functions as a hub that modulates the weighting of information processing through salience attribution within the reciprocal interactions between internal predictions and sensory information. Inferential updating processes that continuously correct mismatches between internal predictions and sensory information are considered to operate within the frontal–striatal–thalamic circuit. These processes can be viewed as one aspect of the interactions between internal representations and external events considered in relation to subjective experience in the present review (**Figure 1**). Aberrant salience attribution arising from dysfunction of this circuit is hypothesized to disturb inferential processing, thereby disrupting the updating between internal models and latent states (**Figure 2**). Such disturbances are proposed to progressively alter subjective experience and contribute to disturbances of subjective consciousness associated with schizophrenia.

Fletcher and Frith (20) proposed that hallucinations and delusions may arise from a common disturbance in hierarchical Bayesian inference, involving abnormal prediction-error-dependent updating of perception and beliefs. Within computational models based on the free-energy principle and predictive processing, two major hypotheses, the strong-prior hypothesis and the weak-prior hypothesis, have been proposed regarding abnormalities in the precision of prior beliefs.

The strong-prior hypothesis suggests that excessively weighted prior beliefs dominate perception and may give rise to hallucinations and other abnormal perceptual experiences. Corlett et al. (21) discussed these alterations in predictive processing as potential mechanisms underlying abnormal perception and belief formation in psychosis. These computational accounts are also supported by empirical studies that directly examined inferential processing using behavioral measures. Powers et al. (22), using a perceptual conditioning task and computational modeling, demonstrated that hallucination-prone individuals showed increased weighting of perceptual priors. Behavioral studies of belief updating have also demonstrated altered updating of beliefs under uncertainty in schizophrenia (23).

In contrast, Sterzer et al. (25) discussed the weak-prior hypothesis, according to which reduced precision of prior beliefs increases the relative weighting of sensory information, resulting in excessive prediction errors and unstable belief updating. From a complementary temporal perspective, Takei et al. (24) proposed the Hyperlearning Hypothesis, in which impaired precision of prediction-error signaling may initially produce unstable perceptual inference, while subsequent maladaptive overlearning may progressively stabilize inaccurate internal models. Although the strong- and weak-prior hypotheses appear contradictory, Adams et al. (26) proposed that predictive processing is organized hierarchically rather than operating at a single computational level. According to this perspective, prior precision may be increased at higher hierarchical levels while being reduced at lower levels. Thus, the strong-prior and weak-prior hypotheses are not necessarily mutually exclusive but may instead reflect abnormalities occurring at different levels of the inferential hierarchy.

This, in turn, raises the important question of when and how abnormalities in hierarchical inferential processing emerge during the course of psychosis. At the neurobiological level, the present review proposes that hierarchical inferential processing may be supported, at least in part, by interactions within the frontal–striatal–thalamic circuit.

Within this model, disturbances in these interactions may disrupt inferential updating between internal representations and external events, thereby contributing to abnormalities in subjective experience. In the following sections, we review structural and functional alterations in the frontal–striatal–thalamic circuit in ARMS/APS, clinical high-risk states for psychosis in which disturbances of subjective experience may already emerge. We then develop a more specific model linking inferential processing to alterations within its subcircuits and address the falsifiability of the proposed model.

## 8 Changes in the frontal lobe in ARMS/APS

Within the frontal–striatal–thalamic circuit, the frontal lobe is considered to contribute to the generation of higher-order predictive signals regarding the external environment based on internal models. Diverse structural and functional alterations in the frontal lobe have been reported in schizophrenia and are conceptualized as hypofrontality. Frontal white matter consists of neural fiber bundles connecting the frontal cortex with the striatum, thalamus, and other cortical regions, thereby constituting the structural basis of large-scale neural information networks. Structural brain alterations have also been observed in individuals at clinical high risk for psychosis. A meta-analysis of individuals at clinical high risk for psychosis identified prefrontal gray matter reductions, including alterations in the inferior frontal region associated with subsequent transition to psychosis (27). These findings raise the further question of whether such early structural alterations also involve white matter pathways connecting distributed brain regions during the clinical high-risk stage.

To further examine frontal white matter alterations in ARMS, we previously reported a single-center, one-year longitudinal DTI study including 41 individuals with ARMS and 16 healthy controls, using a 1.5-T MRI scanner and tract-based spatial statistics (TBSS) with family-wise error (FWE) correction for multiple comparisons. Compared with healthy controls, the ARMS group showed significantly reduced FA in a cluster involving the genu and body of the corpus callosum (FWE-corrected p < 0.05). These findings suggest vulnerability of frontal white matter underlying interfrontal neural connectivity. Such vulnerability may impair information processing networks, potentially contributing to the attenuated symptoms observed in ARMS. Subsequent large-scale and systematic evidence has reported findings broadly consistent with our earlier observations. In schizophrenia, the Enhancing NeuroImaging Genetics through Meta-Analysis (ENIGMA) Schizophrenia diffusion tensor imaging (DTI) meta-analysis showed widespread fractional anisotropy (FA) reductions, including the anterior corona radiata and corpus callosum (28). A later systematic review also showed altered trajectories of white matter maturation in individuals at genetic and clinical high risk for psychosis, including longitudinal alterations in the corpus callosum, although the findings remained heterogeneous (29). In addition, in our longitudinal study, among individuals with ARMS who did not transition to psychosis during the one-year follow-up period (ARMS-N group), increased FA values in white matter regions surrounding the anterior corpus callosum were significantly correlated with improvement in subthreshold positive symptoms (n = 26, r = −0.664, p < 0.001). These findings suggest that plastic changes in frontal white matter networks may be associated with clinical improvement during the follow-up period (30). However, these findings should be regarded as exploratory and hypothesis-generating rather than confirmatory. Non-transition at one year does not necessarily indicate long-term resilience, as ARMS/APS is clinically dynamic and later transition to psychosis may occur (31). Regression to the mean and potential clinical and developmental confounders should also be considered.

## 9 Changes in striatal subregions in ARMS/APS

Within the frontal–striatal–thalamic circuit, the striatum functions as a hub that regulates and processes information circulating between the frontal lobe and the thalamus. The striatum is subdivided into the nucleus accumbens, putamen, and caudate nucleus, each forming distinct frontal–striatal–thalamic subcircuits. These striatal subregions receive inputs from different frontal cortical regions and contribute to dorsolateral prefrontal, orbitofrontal, anterior cingulate (limbic), motor, and oculomotor loops, supporting distinct aspects of executive, emotional, motivational, motor, and oculomotor function (13, 15). These striatal subregions exhibit differential volumetric alterations not only in schizophrenia but also in ARMS/APS (32).

In a previous study, we longitudinally investigated volumetric changes in striatal subregions in individuals with ARMS and healthy controls (33). Structural MRI was acquired using a 1.5-T scanner in 42 individuals with ARMS and 16 healthy controls, and striatal subregional volumes were quantified using FreeSurfer. Longitudinal changes were analyzed using repeated-measures ANOVA and multiple regression analyses. In the ARMS-N group, increases in right nucleus accumbens volume over the one-year follow-up period were significantly associated with improvement in subthreshold positive symptoms, and this association remained significant in the regression model (β = −0.64, p < 0.001; adjusted R² = 0.490). The ventral frontal-nucleus accumbens-thalamic subcircuit, in which the nucleus accumbens functions as a hub, is referred to as the ventral loop. This loop is involved in emotional regulation and reward-related processing, including reinforcement learning and extinction learning (34). Through these processes, the ventral loop may evaluate the behavioral value of information derived from the environment and contribute to value-based selection and adaptive learning (35), thereby facilitating the refinement of internal models. At the structural level, large-scale ENIGMA studies have demonstrated reduced nucleus accumbens volume in schizophrenia (36), whereas the longitudinal increase observed in our ARMS-N group was associated with clinical improvement. This contrasting pattern suggests that nucleus accumbens alterations may vary across clinical stages and trajectories and may be associated with processes of adaptive plasticity. Importantly, frontal–striatal–thalamic subcircuits are not completely segregated but interact through overlapping and integrative territories within the striatum (15).

In contrast, in the same longitudinal study, the ARMS-N group showed a significant longitudinal reduction in total volume of the putamen over one year (effect of time: F (1, 32) = 7.957, p = 0.008). Similar reductions in putaminal volume have been reported in first-episode schizophrenia patients (37). The putamen forms the premotor cortex–putamen–thalamic circuit, a subcircuit of the frontal–striatal–thalamic circuit. This circuit, in which the putamen functions as a hub, is traditionally regarded as a sensorimotor loop receiving inputs from motor and somatosensory cortical regions, including the primary motor cortex, premotor cortex, supplementary motor area, and somatosensory cortex. More recently, this circuit has been implicated not only in motor program selection and regulation but also in psychomotor function, sensorimotor integration, and behavioral selection (35). These functions may be interpreted from the perspective of active inference, in which perception and action are regarded as inseparable processes involved in minimizing prediction error.

Within this subcircuit, the premotor cortex is involved in action planning, observation of others’ behavior, and motor execution. Dysfunction of this region is known to cause apraxia (38) and has been implicated in catatonia and psychomotor symptoms observed in schizophrenia and related psychiatric disorders (39, 40). The premotor cortex is also recognized as a key component of the mirror neuron system (41). Dysfunction of this system has been suggested to contribute to echophenomena (42) and may be associated with disturbances of self–other boundaries and impairments in social cognition (43).

Taken together, these findings suggest that the premotor cortex–putamen–thalamic subcircuit may constitute one neural substrate for active inference related to sensorimotor integration, psychomotor function, and self–other integration. Dysfunction within this subcircuit may contribute to instability in self–other integration and misinterpretation of environmental context. These alterations lead to psychomotor and behavioral abnormalities, thereby contributing to alterations in subjective experience associated with schizophrenia spectrum conditions.

## 10 Changes in the premotor cortex–putamen–thalamic circuit in ARMS/APS

Although our previous studies identified structural alterations in both the nucleus accumbens and putamen in ARMS/APS (33), these findings cannot be directly equated with dopaminergic abnormalities. In PET studies, presynaptic dopaminergic abnormalities in psychosis are more prominent in the dorsal striatum, particularly in the associative striatum, including the dorsal caudate and dorsal putamen, with less prominent abnormalities reported in the ventral striatum, which includes the nucleus accumbens (44–46). Previous studies of catatonia in schizophrenia spectrum disorders have reported white matter abnormalities involving thalamo-premotor and striatal connectivity within the premotor cortex–putamen–thalamic circuit (47). However, it is unclear whether such white matter alterations are present during the prodromal stage of psychosis. These findings provide a rationale for further examining putamen-related subcircuits in the pathophysiology of psychosis.

In a previous study, we therefore examined whether alterations in the premotor cortex–putamen–thalamic circuit were already present in individuals with ARMS/APS before the onset of psychosis. We analyzed FA values in the white matter pathways connecting the premotor cortex with the striatum (ST-PREM) and thalamus (T-PREM) in 36 individuals with APS and 38 healthy controls using diffusion tensor imaging and TractSeg. Logistic regression adjusted for age and sex demonstrated that lower FA values in the right T-PREM were significantly associated with APS status (odds ratio = 1.76, 95% CI: 1.10–2.82, p = 0.02), whereas no significant associations were observed for the ST-PREM. These results suggest that dysfunction of the premotor cortex–thalamic circuit may emerge during the APS stage and subsequently involve the premotor cortex–striatal circuit (48). These findings are consistent with recent pathophysiological models of schizophrenia, proposing that disruption of higher-order control mechanisms within frontal cortical–thalamic system destabilizes functional equilibrium within these circuits and secondarily induces striatal dopaminergic dysfunction (49). Alterations in this circuit may contribute to aberrant inferential processing, thereby disrupting sensorimotor integration, psychomotor function, and social cognition. Such alterations may contribute to distortions in subjective experience associated with prodromal symptoms observed in ARMS/APS. Because the premotor cortex–putamen–thalamic circuit occupies a relatively posterior position within the hierarchical organization of frontal–striatal–thalamic subcircuits, dysfunction within this circuit may destabilize higher-order prefrontal processing through disturbances in more fundamental levels of information processing (50).

## 11 Oculomotor biomarkers related to superior thalamic radiation

The preceding sections described alterations in neural networks in ARMS/APS. Such alterations may disrupt predictive and inferential processing, which may be reflected in behavioral and neurophysiological measures. Mismatch negativity (MMN), an electrophysiological marker of auditory prediction-error processing, is reduced across the psychosis spectrum, including in individuals at clinical high risk for psychosis (51, 52). Rodriguez-Sanchez et al. (53) combined MMN and P300 event-related potentials (ERPs) with biophysical modeling in a clinical high-risk cohort and found reduced ERP amplitudes in individuals who subsequently developed psychosis, best explained by reduced pyramidal cell excitability. Sensory attenuation paradigms provide behavioral measures of the prediction of sensory consequences of self-generated actions and the integrity of forward models (54). Related experimental evidence indicates that both real and imagined sensory feedback can influence action anticipation (55).

Among sensory modalities, visual processing provides another domain for examining predictive processing and active sensory sampling. Saccadic prediction and exploratory eye movements may provide behavioral measures of these processes. Because the eyes function not only as sensory organs but also as motor organs, exploratory eye movements may reflect active inference involving sensorimotor coupling (56). Through this process, the brain actively samples sensory information from the environment to minimize mismatches between internal predictions and sensory inputs. Among the frontal–striatal–thalamic subcircuits, the frontal eye field-caudate nucleus-thalamic circuit constitutes an oculomotor subcircuit specifically involved in eye movement control. Disturbances of exploratory eye movements have been reported not only in schizophrenia but also in APS (57), although the underlying neural mechanisms have not yet been fully elucidated. Abnormal exploratory eye movements in patients with schizophrenia have been repeatedly linked to functional abnormalities in the posterior frontal regions, including the frontal eye field and premotor cortex, as well as in the thalamus. We have previously reported significant structural alterations in the pathways connecting the premotor cortex and thalamus in individuals with APS (48). The frontal eye field and the premotor cortex are adjacent regions within the posterior frontal cortex and are functionally interconnected. We therefore hypothesized that abnormalities in exploratory eye movements in APS would be associated with alterations in the superior thalamic radiation (STR), which connects the posterior frontal regions with the thalamus.

To examine this hypothesis, we conducted a DTI study of 21 individuals with APS and 30 healthy controls, using TractSeg to evaluate FA values of the bilateral STR and an exploratory eye movement test to assess eye movement parameters. The APS group showed significantly reduced FA values in the left STR (age- and sex-adjusted p = 0.021). Notably, FA values in the bilateral STR were significantly correlated with mean eye scanning length (MESL) (left: r = 0.567, p = 0.007; right: r = 0.756, p < 0.001) (58). These findings suggest that microstructural alterations in the STR, a component of the frontal–striatal–thalamic loop, may contribute to abnormalities in exploratory eye movements associated with active sampling in APS. Accordingly, alterations in the STR may represent a potential biomarker of altered inferential processing during the transition from prodromal states to schizophrenia spectrum disorders. From this perspective, alterations in exploratory eye movements may represent one behavioral manifestation of disturbances in the reciprocal interactions between internal representations and external events underlying the emergence of subjective experience (**Figure 1**).

## 12 Discussion

Several biological hypotheses of schizophrenia have been proposed, including the dopamine, hypofrontality, and dysconnectivity hypotheses. Each of these may be understood as reflecting one aspect of dysfunction within the frontal–striatal–thalamic circuit. From a psychopathological perspective, schizophrenia emerges fundamentally through disturbances in subjective consciousness. Husserl (6) emphasized the intentional structure of consciousness, while Jaspers described disturbances of self-consciousness involving identity, unity, boundaries, and agency in psychopathology. These concepts show theoretical continuity with the ipseity or minimal-self disturbance articulated by Sass and Parnas (5), which Nelson et al. (59, 60) further linked to neurocognitive and neuroscientific models. Because its essential pathology belongs to the dimension of subjective phenomena, there are inherent limitations in explaining schizophrenia solely through inductive approaches based on objective indicators, including biological factors. Therefore, in this review, we examined whether phenomenology, which investigates subjective consciousness through deductive approaches, can be theoretically bridged with computational neuroscience based on inductive approaches. These two approaches share a theoretical point of contact, in that both attempt to explain mental activity through inferential processes arising from interactions between internally generated representations and information derived from the external environment. In this review, inferential processing is considered one component of broader information processing and refers specifically to the processes through which internal models are updated by integrating information derived from the external environment. The frontal–striatal–thalamic circuit is proposed as one neural substrate underlying these inferential processes. Dysfunction of this circuit is hypothesized to contribute to maladaptive inferential processing underlying the updating between internal models and latent states. Such processes are proposed to progressively distort subjective experience, thereby contributing to disturbances of subjective consciousness in schizophrenia.

In our previous studies, we identified neurobiological alterations involving multiple components of the frontal–striatal–thalamic circuit in individuals with ARMS/APS, including frontal white matter, striatal subregions, premotor-related pathways, and the superior thalamic radiation. Importantly, these findings were broadly consistent with independent evidence from studies published subsequent to our reports, including large-scale and longitudinal investigations of frontal white matter, striatal, and thalamic abnormalities across the psychosis spectrum. In addition to this accumulating neurobiological evidence, our finding that exploratory eye-movement abnormalities were associated with alterations in the STR suggests that circuit-level alterations may have measurable behavioral correlates.

Taken together, these findings suggest that alterations involving multiple components of the frontal–striatal–thalamic circuit are already present during the clinical high-risk stage, providing an empirical basis for formulating testable predictions regarding their relationship with computationally defined inferential processing and emerging psychopathology. Notably, several of our findings converge on alterations involving posterior frontal cortical–thalamic pathways. These alterations are also broadly consistent with findings from subsequent independent studies.

One possibility is that these posterior frontal cortical–thalamic alterations may disrupt the balance of reciprocal interactions underlying circular causality, thereby disturbing precision weighting both within and across levels of the inferential hierarchy. Such disruption could potentially produce weak-prior abnormalities at some hierarchical levels and strong-prior abnormalities at others. These hierarchical imbalances may also contribute to alterations in striatal processing. However, because frontal, striatal, and thalamic abnormalities have each been reported before psychosis onset, their temporal relationships remain to be established. Importantly, our neuroimaging studies did not include direct measures of dopaminergic function, MMN, or computational estimates of precision weighting. Therefore, the observed structural changes cannot directly establish weak- or strong-prior abnormalities within specific components of the frontal–striatal–thalamic circuit. Regarding the falsifiability of this model, if alterations in precision weighting are consistently found to occur independently of functional and structural abnormalities within the frontal–striatal–thalamic circuit, the proposed model would need to be reconsidered.

Although dysfunction of the frontal–striatal–thalamic circuit has primarily been investigated as a pathophysiological basis of schizophrenia, recent studies have also implicated this circuit in various psychiatric disorders, including mood disorders and attention-deficit/hyperactivity disorder (ADHD) (19). More broadly, large-scale transdiagnostic studies have identified shared structural and functional neural abnormalities across multiple psychiatric disorders (61, 62). At the same time, ARMS/APS has been shown to progress not only to schizophrenia but also to mood disorders and personality disorders (63, 64). Accumulating transdiagnostic findings therefore suggest the need to reconsider dysfunction of the frontal–striatal–thalamic circuit not as a schizophrenia-specific abnormality, but rather as a transdiagnostic neural mechanism. Such dysfunction may underlie disturbances in subjective experience across the spectrum of Clinical High At Risk Mental States (CHARMS) through abnormalities in inferential processing between internal models and latent states. These observations raise the possibility that psychopathological phenomena traditionally discussed separately across psychiatric disorders may partially reflect common disturbances in inferential processing. Future transdiagnostic studies encompassing neuroimaging, computational modeling, and phenomenological approaches will be necessary to further clarify these inferential processes and their relationship to disturbances in subjective experience across psychiatric disorders.

## 13 Limitations and future perspectives

Several limitations should be acknowledged in this review.

First, the review focused on the frontal–striatal–thalamic circuit because it is implicated in inferential processing between internal representations and external events, thereby providing a theoretical point of contact between phenomenology and computational neuroscience. However, the frontal–striatal–thalamic circuit should be considered as one component of broader neural systems involved in inferential processing, rather than as a unitary or exclusive substrate. Its multiple interacting subcircuits have distinct but overlapping functions (15), and temporal, parietal, and cerebellar–thalamic–cortical systems may also contribute to inferential processing in psychosis (65). Although several components of the proposed model are consistent with converging evidence from previous studies, our longitudinal neuroimaging findings were derived from single-center cohorts with relatively modest sample sizes. These findings should therefore be regarded as hypothesis-generating and require replication in larger, independent cohorts using complementary approaches.

A second limitation is that many of the findings concerning the frontal–striatal–thalamic circuit discussed in this review primarily involve subcircuits centered on the posterior frontal regions, whereas relatively few findings are available regarding subcircuits associated with anterior frontal regions. However, this imbalance does not reflect a deliberate focus on the posterior frontal region. Within hierarchically organized information-processing systems, increasing hierarchical levels are associated with increasingly integrative and abstract information processing (11). In the brain, the posterior frontal regions occupy lower hierarchical levels and are involved in concrete and observable information processing that is amenable to mathematical and neuroscientific investigations. Accordingly, regions located closer to the frontal pole are thought to process information spanning broader temporal and contextual scales based on the integration of information derived from multiple lower hierarchical levels. Such information processing is inherently integrative and abstract, rendering direct mathematical and neuroscientific investigations increasingly difficult (50).

Third, this limitation of quantitative characterization arising from hierarchical organization becomes particularly important when considering human subjective experience. Human subjective experience is not confined to ongoing mechanisms of inferential processing at a given point in time, but emerges through multiple interacting processes that are continuously organized into hierarchical structures over time. As these hierarchical structures develop through the progressive integration of information, the processes contributing to subjective experience become increasingly complex and abstract, incorporating information across broader temporal and contextual scales. Beyond quantitative descriptions, Heidegger (66), in *Being and Time* (German: *Sein und Zeit*), emphasized the fundamental relationship between human existence and temporality, and Fuchs examined disturbances of temporality in schizophrenia from the perspective of phenomenological psychopathology (67). In clinical psychiatric and psychological practice, understanding an individual’s life history as shaped by subjective temporality is a fundamental aspect of the therapeutic process. Thus, computational and neurobiological accounts of inferential processing should not be regarded as substitutes for phenomenological and narrative approaches to the temporal and biographical dimensions of subjective experience, which remain central to clinical psychiatric and psychological practice and therapeutic care (68).

## 14 Conclusion

In this review, we reconsidered schizophrenia, traditionally understood in psychopathology as a disorder of subjective consciousness, from the perspective of information processing. Alterations in the frontal–striatal–thalamic circuit reported in ARMS/APS, including our own findings, were consistent with the hypothesis that instability in corrective processes for discrepancies between internal representations and external events contributes to the formation of psychotic experiences.

The proposed model generates testable predictions regarding how the relationships among frontal cortical–thalamic and striatal alterations, abnormal precision weighting, and corresponding perceptual and behavioral changes may vary across stages of psychosis. At a conceptual level, this model provides a theoretical point of contact between phenomenological and computational approaches by linking neural activity, perceptual and behavioral alterations, and subjective experience. This perspective may contribute to the development of novel transdiagnostic concepts and biomarkers reflecting early disturbances in information processing across conventional diagnostic boundaries. Future studies integrating neuroimaging, computational, behavioral, and phenomenological approaches will be required to test these predictions and further evaluate the proposed model, with the ultimate goal of improving the early identification and clinical understanding of individuals at risk for psychosis and informing more individualized therapeutic approaches.
